# Occupational heat protection policies

**DOI:** 10.2471/BLT.26.295639

**Published:** 2026-07-01

**Authors:** Xin Zhou, Shi Huang, Xinman Hu, Weiyi Xu, Yidu Zheng, Hongyuan Li, Haitao Zhao, Xuemei Zhang, Zhen Pu, Ruixiang Li

**Affiliations:** a The Affiliated Hospital of Southwest Medical University, Luzhou, China.; b Clinical Medical College, Southwest Medical University, No. 319, Section 3, Zhongshan Road, Jiangyang District, Luzhou, Sichuan 646000, China.; c Chengdu College of University of Electronic Science and Technology of China, Chengdu, China.

Recent international calls for action have identified extreme heat as a serious public health risk and have reinforced the need for national and subnational heat action plans.^1^ However, many plans still place more focus on heat alerts, public information and preparedness of health-care systems than on worksites where exposure is predictable and often sustained. A heat warning has limited protective value for a farm worker, construction labourer, delivery rider or market trader who cannot stop work without losing income. Heat action plans should therefore make workplace heat protection a core test of preparedness, equity and accountability.

The World Health Organization (WHO) and the World Meteorological Organization (WMO) have reported that billions of workers are exposed to high temperatures, with risks concentrated among people engaged in manual labour outdoors or in hot indoor environments.^2^ The International Labour Organization (ILO) reports similarly emphasize that climate change is already affecting occupational safety and health, while legal provisions and enforcement systems have not kept pace with the changing risk.^3^^,^^4^ Given this evidence, we suggest that heat action plans move beyond general recognition of occupational heat risk and specify who is most exposed, which authority is responsible for prevention, what minimum protections are required and how governments will verify that workers have actually received them. 

Building on these reports,^3^^,^^4^ we propose several operational recommendations for making occupational heat protection more visible in heat action plans. Heat action plans should include occupational indicators, because plans measured only by warnings issued, websites updated or health-care facilities alerted may overstate preparedness while exposed workers still lack basic protections such as water, rest, shade, schedule adjustment, acclimatization support or safe referral. Governments should treat workplaces as settings of heat risk, in the same way as they do with schools, care homes and housing in other public-health contexts. Plans should therefore distinguish workplace-specific monitoring indicators from minimum protective requirements. Monitoring indicators could include mapping high-risk sectors before the heat season, deploying labour inspectors during alerts and reporting work-related heat illness quickly enough to guide action. Minimum requirements could include requiring employers to adjust tasks, schedules and rest arrangements when predefined heat thresholds are reached. In these plans, high-priority sectors should include agriculture and construction. Agricultural workers face heavy physical demands, prolonged sun exposure, long working hours, piece-rate incentives and often limited access to drinking water or cool-down areas. Construction workers face outdoor heat, heavy protective equipment, deadline pressure and radiant heat from sun-exposed roofs, roads, walls and machinery. These sectors are policy sentinels because if a heat action plan cannot protect workers in these settings, its protective reach is likely to be weak elsewhere. The workplace heat-protection focus, however, should not stop with these two sectors. Sanitation workers, street vendors, transport workers, delivery riders, market traders, security staff, fishing and mining workers, domestic workers and factory workers in poorly ventilated spaces may all face dangerous heat exposure.^2^^–^^5^

Equity must be a core operational priority because heat at work often affects people with the least power to decide when, where and how they work. The ILO recognizes that a safe and healthy working environment is a fundamental principle and right at work, providing a normative anchor for heat policy.^6^ The purpose of heat policy should not be to preserve productivity alone, but to prevent avoidable illness, injury, income loss and death among workers whose bargaining power is limited.

Women need to be more visible in workplace heat-risk assessment and planning. Women may be undercounted if plans define hot work solely as male-dominated construction or heavy industry. Women work in agriculture, markets, domestic service, waste collection, food preparation, garment work and home-based production, often informally and with limited social protection. The Food and Agriculture Organization has documented unequal climate impacts on the rural poor, women and young people.^7^ Gender-sensitive heat protection requires safe access to water, toilets and rest areas; attention to pregnancy, menstrual hygiene and harassment risks; and recognition of combined paid work and unpaid care.

Informal workers need a different implementation path. In many settings, relying only on obligations placed on formal employers will leave many exposed informal workers outside protection. Local governments can provide water points, shaded rest areas and cooling access in markets, transport hubs, agricultural collection points and industrial clusters. Community organizations, worker associations and municipal services can help communicate heat warnings and link workers to non-punitive complaint and referral systems. Workers should not face job loss, wage loss or retaliation for reporting symptoms, requesting rest or stopping unsafe work.

Heat action plans should assign institutional responsibilities before the hot season. Labour authorities are best placed to regulate employers, define enforceable duties, inspect worksites and apply sanctions where prevention fails. Health authorities should define health-relevant thresholds, prepare emergency and primary health-care services, issue clinical guidance, integrate work-related heat illness into surveillance and feed data back to labour authorities and local governments.^2^^,^^8^ Governments should include meteorological services, social protection agencies and municipalities in pre-season planning, rather than improvising their roles during an alert. Without such arrangements, heat plans can become warnings without enforcement capacity.

Urban governance also shapes workplace heat protection. WHO’s healthy cities approach emphasizes health, equity and multisectoral urban action,^9^ and WHO guidance on heat-health action planning sets out principles for preventing and responding to heat-related risks.^10^ For workers, urban heat is shaped by shade, housing, transport, public space, water access, municipal contracting and neighbourhood vulnerability. For example, the heat action plan of Ahmedabad, India, links colour-coded heat alerts with a municipal nodal officer, interagency communication, public outreach, training for health workers, activation of cooling centres and longer-term cool-roof measures. The plan also includes actions relevant to workers, such as identifying outdoor workers as a vulnerable group and encouraging measures to reduce heat exposure during severe alerts.^11^ Heat action plans should therefore connect workplace protection with city cooling strategies instead of treating labour, transport, housing and public-space policy as separate domains.

Governments should make minimum workplace protections enforceable commitments in heat action plans. When heat exceeds predefined thresholds, employers should ensure cool drinking water, rest breaks, shaded or cooled recovery spaces, workload and schedule adjustment, acclimatization for new or returning workers, training on early symptoms and referral or emergency response for suspected illness.^2^^–^^5^^,^^8^ Indoor workplaces should improve ventilation, reduce radiant heat or redesign processes where feasible. These requirements should be adapted to local conditions and capacity, but they should not be reduced to voluntary advice.

Heat action plans can use monitoring approaches that match local capacity. High-capacity systems can use emergency visits, ambulance dispatches, compensation claims and occupational injury notifications during heat events. Lower-resource systems can use sentinel reporting from selected emergency departments, primary health-care facilities, occupational clinics or district hospitals, combined with rapid end-of-day summaries from local health offices. The goal is not perfect surveillance but intelligence fast enough to trigger outreach, clinical readiness, municipal cooling and labour inspection while the heat event is still unfolding.

Accountability should be close to the site of exposure. Occupational safety and health law, labour inspection, procurement standards, employer records, workers’ compensation, collective bargaining and public disclosure are more direct accountability tools than generic preparedness scores. The United States Occupational Safety and Health Administration’s *National emphasis program for outdoor and indoor heat-related hazards* illustrates an operational model by linking heat-related hazards to targeted inspection and enforcement activities.^12^ Other heat action plans should adopt the same implementation logic, that is, thresholds should trigger duties, duties should be inspectable and failures should lead to corrective action.

National and subnational heat action plans will remain insufficient if they only include alerts and recommendations. These plans should identify workers as a high-risk group; give special attention to agriculture, construction and other high-exposure sectors; extend protection to women, migrants and informal workers; assign enforcement to labour authorities and surveillance to health systems; and connect workplace protection with urban heat governance. Extreme heat is an environmental emergency as well as a test of whether governments can protect the people whose labour supports food systems, cities and essential services in a warming world.
